# Supplementary material for: Mental health of vulnerable groups experiencing a drought or bushfire: A systematic review
Source: Glob Ment Health (Camb). 2023 Apr 13;10:e24. doi: 10.1017/gmh.2023.13 (PMC10581865; doi:10.1017/gmh.2023.13)
Supplement: Supplementary file 1 [file S2054425123000134sup001.docx]

**Supplementary Table 1: Joanna Briggs Quality Assessment for qualitative studies included in the review**

| First Author (Year) – Country | 1 | 2 | 3 | 4 | 5 | 6 | 7 | 8 | 9 | 10 | Total Score |
| --- | --- | --- | --- | --- | --- | --- | --- | --- | --- | --- | --- |
| **Humphreys (2022) - USA** | Yes | Yes | Yes | Yes | Yes | Yes | Yes | Unclear | Yes | Yes | 9/10 |
| **Hayati (2010) - Iran** | Yes | Yes | Yes | No | Yes | No | No | Yes | No | Yes | 6/10 |
| **Hossain (2008) - Australia** | No | Yes | Yes | Yes | Yes | No | No | Yes | Yes | Yes | 7/10 |
| **Sartore (2008) - Australia** | Yes | Yes | Yes | Yes | Yes | No | No | Yes | Yes | Yes | 8/10 |
| **Rigby (2011) - Australia** | Yes | Yes | Yes | Yes | Yes | No | No | Unclear | No | Yes | 6/10 |
| **Dominguez (2022) - USA** | Yes | Yes | No | Yes | Yes | Yes | Yes | Unclear | Yes | Yes | 8/10 |

Joanna Briggs assessment criteria for qualitative studies:

1. Is there congruity between the stated philosophical perspective and the research methodology?
2. Is there congruity between the research methodology and the research question or objectives?
3. Is there congruity between the research methodology and the methods used to collect data?
4. Is there congruity between the research methodology and the representation and analysis of data?
5. Is there congruity between the research methodology and the interpretation of results?
6. Is there a statement locating the researcher culturally or theoretically?
7. Is the influence of the researcher on the research, and vice- versa, addressed?
8. Are participants, and their voices, adequately represented?
9. Is the research ethical according to current criteria or, for recent studies, and is there evidence of ethical approval by an appropriate body?
10. Do the conclusions drawn in the research report flow from the analysis, or interpretation, of the data?

**Supplementary Table 2.** **Joanna Briggs Quality Assessment for cross-sectional studies included in the review**

| First Author (Year) – Country | 1 | 2 | 3 | 4 | 5 | 6 | 7 | 8 | Total score |
| --- | --- | --- | --- | --- | --- | --- | --- | --- | --- |
| **Papanikolaou, Adamis (2011) - Greece** | Yes | No | Yes | Yes | Yes | Yes | Yes | Yes | 7/8 |
| **Scher (2009) - USA** | No | Yes | Yes | Yes | No | No | Yes | Yes | 5/8 |
| **Stain (2011) - Australia** | Yes | Yes | Yes | Yes | Yes | Yes | No | No | 6/8 |
| **Cowlishaw (2021) - Australia** | Yes | Yes | Yes | Yes | Yes | Yes | Yes | Yes | 8/8 |
| **Belleville (2021) - Canada** | Yes | Yes | Yes | No | Unclear | Yes | Yes | Yes | 6/8 |
| **Friel (2014) - Australia** | Yes | Yes | Yes | Yes | Yes | Yes | Yes | Yes | 8/8 |
| **Papanikolaou, Leon (2011) - Greece** | Yes | Yes | Yes | No | No | No | Yes | No | 4/8 |
| **Kelly (2011) - Australia** | Yes | Yes | Yes | Yes | Unclear | Unclear | Yes | Yes | 6/8 |

Joanna Briggs assessment criteria for cross-sectional studies:

1. Were the criteria for inclusion in the sample clearly defined?
2. Were the study subjects and the setting described in detail?
3. Was the exposure measured in a valid and reliable way?
4. Were objective, standard criteria used for measurement of the condition?
5. Were confounding factors identified?
6. Were strategies to deal with confounding factors stated?
7. Were the outcomes measured in a valid and reliable way?
8. Was appropriate statistical analysis used?

**Supplementary Table 3. Joanna Briggs Quality Assessment for cohort studies included in the review**

| First Author (YEAR) - Country | 1 | 2 | 3 | 4 | 5 | 6 | 7 | 8 | 9 | 10 | 11 | Total score |  |
| --- | --- | --- | --- | --- | --- | --- | --- | --- | --- | --- | --- | --- | --- |
| **Austin (2018) – Australia** | Yes | Yes | Yes | Yes | Yes | Unclear | Yes | Yes | No | Unclear | Yes | 8/11 |  |
| **Carroll (2022) – Australia** | Yes | Yes | No | Yes | Yes | Unclear | Yes | Yes | Yes | Unclear | Yes | 9/11 |  |
| **Parslow (2006) – Australia** | Yes | Yes | No | No | No | Unclear | Yes | Yes | No | No | Yes | 5/11 |  |
| **Powers (2015) - Australia** | Yes | Yes | Yes | Yes | Yes | No | Yes | Yes | Yes | Yes | Yes | 10/11 |  |

Joanna Briggs assessment criteria for cohort studies:

1. Were the two groups similar and recruited from the same population?
2. Were the exposures measured similarly to assign people to both exposed and unexposed groups?
3. Was the exposure measured in a valid and reliable way?
4. Were confounding factors identified?
5. Were strategies to deal with confounding factors stated?
6. Were the groups/participants free of the outcome at the start of the study (or at the moment of exposure)?
7. Were the outcomes measured in a valid and reliable way?
8. Was the follow up time reported and sufficient to be long enough for outcomes to occur?
9. Was follow up complete, and if not, were the reasons to loss to follow up described and explored?
10. Were strategies to address incomplete follow up utilized?
11. Was appropriate statistical analysis used?
